# Removal of Spinal Instrumentation Is Not Required to Successfully Treat Postoperative Wound Infections in Most Cases

**DOI:** 10.7759/cureus.56380

**Published:** 2024-03-18

**Authors:** Viral Patel, Ben Mueller, Amir A Mehbod, Manuel R Pinto, James D Schwender, Timothy A Garvey, John M Dawson, Joseph H Perra

**Affiliations:** 1 Orthopedic Surgery, Twin Cities Spine Center, Minneapolis, USA; 2 Research, Twin Cities Spine Center, Minneapolis, USA

**Keywords:** revision surgery, implant retention, instrumented spinal fusion, surgical site infection, deep wound infection

## Abstract

Introduction: Controversy exists regarding whether spinal implants need to be removed to treat postoperative deep wound infections (DWIs). This retrospective study aimed to determine whether the removal or retention of implants impacts the successful treatment of a DWI after spine surgery.

Methods: Postoperative spine surgery patients presenting with signs of infection who underwent irrigation and debridement (I&D) at Twin Cities Spine Surgeons at Abbott Northwestern Hospital, Minnesota, USA, were studied. First, the persistence of infection when implants were retained or removed was assessed. Second, we analyzed the persistence of infection with respect to the number of I&D, the use of vacuum-assisted closure (VAC) treatment, pseudoarthrosis status, and functional outcomes.

Results: One hundred thirty-five patients were included. Treatment of infection with retention of implants occurred in 64% (87/135); of these, 7% (6/87) had a persistent infection. Of patients with implant removal (36%, 48/135), 6% (3/48) had a persistent infection. Thus, we observed no difference between treatment with implants present compared to implants removed (p = 1.0). Fifty of the 135 patients (37%) received I&D and primary wound closure, and 85 (63%) patients received I&D and VAC treatment. There was no statistical difference between primary wound closure and VAC treatment (p = 0.15) with respect to persistence. Repeat I&D with VAC (three or more times) had a significantly lower rate of recurrence than those with two I&Ds. Pseudoarthrosis and persistent infection were unrelated. At minimum one-year follow-up, achieving a minimum clinically important difference in functional outcome was independent of persistent infection status.

Conclusion: Persistent infection was unrelated to the retention of implants. When VAC treatment was deemed necessary, more than two I&Ds resulted in a significantly better cure for infection. Those with a persistent infection were no more likely to exhibit pseudoarthrosis than those with no persistent infection. All patients showed improvement in functional outcomes at minimum one-year follow-up.

## Introduction

Implants add to the complexity of treating a deep wound infection (DWI) after spine surgery. Most readmissions for DWIs occur early in the postoperative period [1]. When an infection is diagnosed, some, but not all, recommend removal of implants (ROIs) to prevent recurrence [2,3,4,5]. ROIs before the fusion mass can fully consolidate may result in loss of stability, deformity progression, and/or pseudoarthrosis, all of which may require future revision spine surgery. However, Khanna et al. reported recently that implants may be retained without infection recurrence, which concurs with the Second International Consensus Meeting (ICM) on Musculoskeletal Infection [6,7].

The aim of this study is to determine whether the removal or retention of implants impacts the successful treatment of DWIs in spine surgery. We hypothesized that the persistent infection rate after DWIs is not related to retention or ROIs.

## Materials and methods

Subjects

Patients undergoing irrigation and debridement (I&D) of a prior surgical site (after decompression, decompression, and fusion or fusion procedures) at Twin Cities Spine Surgeons at Abbott Northwestern Hospital, a specialty spine center in Minnesota, USA, over a 10-year period were retrospectively reviewed. Excluded were patients who were under 18, who did not consent to research, who underwent I&D for superficial infection, or who experienced an epidural hematoma or primary epidural abscess. Patients undergoing the index surgery at an outside hospital were also excluded. The symptoms before I&D, the time interval between the index surgery and the first I&D, the number of I&D, and the type of treatment (primary closure vs. vacuum-assisted closure, VAC) were abstracted from the electronic medical records. (VAC treatment uses a synthetic sponge, cut to match the size of the open wound, and applied intermittent negative pressure to close the dead space, clear infection and stimulate wound vascularity [8].) The index surgery was defined as the spine surgery that preceded the infection. Persistent infection was defined as a DWI requiring further I&D after completion of the course of the primary treatment with I&D, antibiotics, and wound closure.

Implant status

Implant status was divided into three categories: non-instrumented, instrumented-retained, and instrumented-removed. Non-instrumented subjects had no implants present at the time of the first I&D, instrumented-retained subjects had instrumentation at the first I&D that was retained, and instrumented-removed subjects had instrumentation at the first I&D that was removed during that procedure.

Radiographic evaluation

Postoperative anterior-posterior and lateral X-rays and computed tomography (CT) scans (if available) were evaluated. Patients who did not have either X-rays or CT scans were excluded from radiographic analyses. Patients who had radiographic or intraoperative evidence of pseudoarthrosis were categorized as having pseudoarthrosis; otherwise, they were placed in the “no identified-pseudoarthrosis” group.

Microbial evaluation and treatment

Pre-I&D wound samples and samples collected at I&D were cultured. Results were usually available three to seven days after the samples were obtained, and findings were evaluated by infectious disease providers in our hospital system. Antibiotics were prescribed in accordance with their recommendations. We did not have an established protocol for antibiotic treatment; patients were treated individually on a case-by-case basis.

Clinical evaluation

Functional status was evaluated using the Oswestry Disability Index (ODI) or Neck Disability Index (NDI) preoperatively and at the last follow-up (a minimum of one year postoperatively relative to the index surgery). Visual analog scales (VASs) were used to quantify back and leg pain or neck and arm pain. Minimum clinically important differences (MCIDs) for all lumbar and thoracic spine surgery patients were calculated between the preoperative and last follow-up scores. Thresholds for MCIDs were 12.8 for ODI, 1.2 for VAS back pain, and 1.6 for VAS leg pain [9].

Statistical analysis

We hypothesized that the persistent infection rate after DWI is independent of the implant status. Chi-square and Fisher exact tests were used for dichotomous variables. For normally distributed continuous variables, the Student’s t-test was used to compare means. The Mann-Whitney test for two independent samples was used otherwise. A two-sided p-value less than 0.05 was required for significance. The data analysis for this paper was generated using the Real Statistics Resource Pack software (Copyright 2013-2023, Charles Zaiontz, www.real-statistics.com). Graphs were created using Microsoft® Excel® for Microsoft 365 MSO (64-bit, version 2309, build 16.0.16827.20278).

Source of funding

This research received no funding from public, commercial, or not-for-profit sectors.

## Results

Subjects

**Figure 1:**
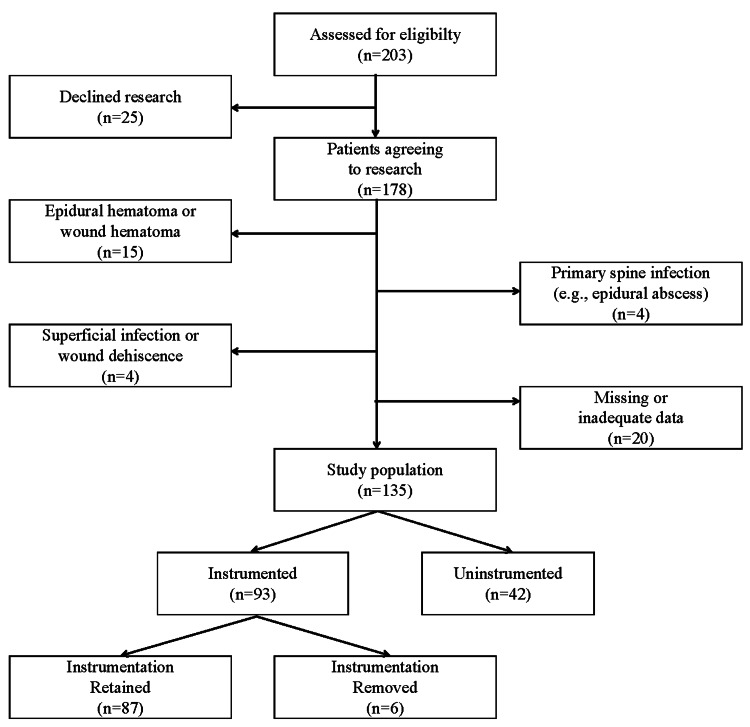
Subject flow diagram

We identified 203 patients who underwent I&D for a spine infection (Figure 1). Sixty-eight (68) patients were excluded. In total, 135 patients (69 males and 66 females) met the inclusion criteria for the study. Patients presented with one or more signs of infection prior to the first I&D, including a draining wound (109/135, 81%), fever (61/135, 45%), pain (47/135, 35%), wound dehiscence with draining (18/135, 13%), altered mental status (18/135, 10%), wound erythema, nausea/vomiting, dyspnea, fatigue, and septicemia (each 1/135, 1%). One patient died from a massive pulmonary embolism postoperatively and one patient died from sepsis and multiple organ system failure. Twelve others died of non-spine surgery-related causes. Available radiographic images represented a median period of 1.7 years after the index surgery (range 0.1 to 11.7 years).

Implant status

Of 135 patients, 42 (31%) were non-instrumented and 93 (69%) were instrumented at the time of the first I&D (Figure 1). The instrumentation was retained in 87 subjects and removed in six. The instrumented-retained group was significantly older than the two other groups (Table 1). Otherwise, the patient characteristics of the three groups were not different. There were no differences in health history except that the instrumented-removed patients had a significantly higher rate of prior methicillin-resistant *Staphylococcus aureus* (MRSA) infection, and there were more ASA 1 patients in the non-instrumented cohort (Table 2). Non-instrumented patients were all 1- or 2-level posterior-approach procedures and mostly decompressions only with no bone grafting (Table 3). Non-instrumented patients experienced less estimated blood loss (EBL), shorter length of surgery, and shorter length of hospital stay.

**Table 1:** Study population characteristics

Variable	All patients (n = 135)	Non-instrumented (n = 42)	Instrumented-retained (n = 87)	Instrumented-removed (n = 6)	p-value
Age (years), mean±SD	55±16	49±15	58±15	49±20	<0.01
BMI, mean±SD	32±8.3	33±8.7	33±8.2	30±7.0	0.81
Female sex, n (%)	66 (49%)	17 (40%)	46 (53%)	3 (50%)	0.42
Smoking at time of surgery, n (%)	31 (23%)	12 (29%)	17 (20%)	2 (33%)	0.43
Workers’ compensation, n (%)	19 (14%)	10 (24%)	8 (9%)	1 (17%)	0.08
Using illicit drugs, n (%)	6 (4%)	2 (5%)	3 (5%)	1 (17%)	0.32

**Table 2:** Study population health history MRSA: methicillin-resistant *Staphylococcus aureus*, ASA: American Society of Anesthesiologists

Variable	All patients (n = 135)	Non-instrumented (n = 42)	Instrumented-retained (n = 87)	Instrumented-removed (n = 6)	p-value
Opioid tolerant, n (%)	36 (27%)	8 (19%)	27 (31%)	1 (17%)	0.30
Comorbidities present, n (%)	52 (39%)	14 (33%)	37 (43%)	1 (17%)	0.32
Prior MRSA infection, n (%)	4 (3%)	1 (2%)	1 (1 %)	2 (33%)	<0.01
Major comorbidities, n (%)					
Diabetes mellitus	24 (18%)	10 (24%)	13 15(%)	1 (17%)	0.47
Chronic kidney disease	4 (3%)	1 (2%)	3 (3%)	0	0.86
Coronary artery disease	18 (13%)	3 (7%)	14 (16%)	1 (17%)	0.36
Chronic obstructive pulmonary disease	11 (8%)	2 (5%)	9 (10%)	0	0.42
Cerebrovascular disease	3 (2%)	1 (2%)	2 (2%)	0	0.93
ASA category					
1	5 (4%)	5 (12%)	0	0	0.04
2	69 (51%)	22 (52%)	43 (50%)	4 (67%)	0.04
3	59 (44%)	15 (36%)	42 (49%)	2 (33%)	0.04
4	1 (1%)	0	1 (1%)	0	0.04
Chronic steroids, n (%)	7 (6%)	1 (2%)	6 (7%)	0	0.47
Immunosuppressants, n (%)	1 (1%)	1 (2%)	0	0	0.33

**Table 3:** Index surgery details

Variable	All patients (n = 135)	Non-instrumented (n = 42)	Instrumented-retained (n = 87)	Instrumented-removed (n = 6)	p-value
Surgical site, n (%)					
Cervical/cervicothoracic	14 (10%)	3 (7%)	11 (13%)	0	0.17
Thoracic/thoracolumbar	21 (16%)	2 (5%)	17 (20%)	2 (33%)	
Lumbar	99 (73%)	37 (88%)	58 (67%)	4 (67%)	
Sacral	1 (1%)	0	1 (1%)	0	
Surgical approach, n (%)					
Posterior only	109 (81%)	42 (100%)	62 (71%)	5 (83%)	<0.01
Anterior only	1 (1%)	0	1 (1%)	0	
Anterior/posterior	25 (19%)	0	24 (28%)	1 (17%)	
Surgical objective, n (%)					
Decompression	50 (37%)	41 (98%)	9 (10%)	0	<0.01
Decompression and fusion	62 (46%)	0	20 (23%)	3 (50%)	
Fusion	23 (17%)	1 (2%)	58 (67%)	3 (50%)	
Revision surgery, n (%)	57 (42%)	12 (29%)	43 (49%)	2 (33%)	0.07
Number of operated levels, n (%)					
1-2	77 (57%)	32 (76%)	42 (48%)	3 (50%)	0.03
3-4	39 (29%)	7 (17%)	31 (36%)	1 (17%)	
5 or more	19 (14%)	3 (7%)	14 (16%)	2 (33%)	
Bone graft used, n (%)					
Autograft	9 (7%)	0	8 (9%)	1 (17%)	<0.01
Allograft	24 (18%)	0	22 (25%)	2 (33%)	
Autograft/allograft	44 (33%)	0	42 (48%)	2 (33%)	
None	58 (43%)	42 (100%)	15 (17%)	1 (17%)	
Estimated blood loss (ml), mean±SD	507±840	115±177	703±980	342±199	<0.01
Length of surgery (min), mean±SD	195±128	97±59	239±129	243±55	<0.01
Length of hospital stay (days), median (range)	4 (1-30)	1 (1-6)	5 (1-30)	5 (3-5)	<0.01
Peri- or post-op complication, n (%)	20 (15%)	5 (12%)	14 (16%)	1 (17%)	0.81

Irrigation and debridement details

Most patients (110) presented with incisional wound drainage; 61 patients presented with fever and 46 patients presented with pain. Culture results from the intraoperative samples produced *Staphylococcus aureus* (*S. aureus*, MRSA or methicillin-sensitive *S. aureus* (MSSA)) in 71/135 (53%) patients, polybacteria in 23 (17%) patients, other bacteria in 10 (7%) patients, and no growth in 20 (15%) patients (Table 4). No intraoperative cultures were collected for three patients, but their infections were clinically confirmed at I&D as were those with no growth of cultures. (It is suspected that these negative results may be due to preoperative treatment with antibiotics.) The instrumented-removed subjects had no MSSA results on culture, but in the other cohorts, MSSA was the primary infective agent. Among our nine subjects with persistent infections, five had MSSA, two had MRSA, one had *Klebsiella pneumoniae* and *Escherichia coli*, and one had *Peptostreptococcus* micros. Only the subject with *K. pneumoniae* and *E. coli* had a prior staph infection. Inflammatory markers were considered for diagnosis of infections when patients presented with wound drainage. Sixty-six (66) patients (49%) received antibiotics before I&D and 69 (51%) patients did not. After the first I&D, all patients received antibiotics for at least four to six weeks through intravenous, oral, or combined routes. 

**Table 4:** Wound culture and antibiotic details MRSA: methicillin-resistant *Staphylococcus aureus*, MSSA: meticillin-sensitive *Staphylococcus aureus*

Variable	All patients (n = 135)	Non-instrumented (n = 42)	Instrumented-retained (n = 87)	Instrumented-removed (n = 6)	p-value
Receiving antibiotics prior to I&D, n (%)	66 (49%)	17 (40%)	45 (52%)	4 (67%)	0.33
Antibiotics prior to 1st I&D, n (%)					
Beta-lactam (cefazolin, ceforoxime)	110 (81%)	34 (78%)	73 (77%)	3 (38%)	0.47
Gylocopeptide (vancomycin)	21 (16%)	6 (13%)	12 (13%)	3 (38%)	
Aminoglycoside (gentamicin)	15 (11%)	5 (11%)	8 (9%)	2 (25%)	
Lincomycin (clindamycin)	1 (1%)	0	1 (1%)	0	
Intraoperative culture result, n (%)					
Coagulase-negative *Staphylococcus*	8 (6%)	2 (5%)	6 (7%)	0	<0.01
*Staphylococcus aureus*	1 (1%)	0	1 (1%)	0	
MRSA	13 (10%)	4 (10%)	6 (7%)	3 (50%)	
MSSA	57 (42%)	23 (55%)	34 (39%)	0	
Polybacteria	23 (17%)	6 (14%)	16 (18%)	1 (17%)	
*Streptococcus*	6 (4%)	1 (2%)	5 (6%)	0	
*Enterococcus*	1 (1%)	0	1 (1%)	0	
*Propionibacterium acnes*	1 (1%)	0	0	1 (17%)	
*Pseudomonas aeruginosa*	1 (1%)	0	1 (1%)	0	
Propionbacterium	1 (1%)	0	1 (1%)	0	
No growth	20 (15%)	5 (12%)	14 (16%)	1 (17%)	
No culture obtained	3 (2%)	1 (2%)	2 (2%)	0	
Received antibiotics after I&D					
IV antibiotics	67 (50%)	17 (40%)	48 (55%)	2 (33%)	0.24
Oral antibiotics	19 (14%)	6 (14%)	13 (15%)	0 (0%)	
Both IV and oral antibiotics	48 (36%)	18 (43%)	26 (30%)	4 (67%)	
Duration of antibiotic treatment after I&D (weeks), median (range)					
IV antibiotics	6 (0.5-12)	4 (1-6)	6 (0.5-12)	6 (6-6)	0.16
Oral antibiotics	2 (1-7)	2 (1-7)	2 (1-4)	0	0.75
Both IV and oral antibiotics	IV: 4 (0.5-8) Oral: 4 (1-17)	4 (0.5-6) 4 (1-12)	4 (0.5-7) 4 (1-12)	6 (1-8) 5.5 (4-17)	0.15 0.10
Antibiotics prescribed after the first I&D, n (%)					
Beta-lactams	97 (45%)	26 (41%)	68 (48%)	3 (30%)	0.02
Quinolones	18 (8%)	9 (14%)	9 (6%)	0 (0%)	
Tetracyclines	9 (4%)	3 (5%)	6 (4%)	0 (0%)	
Sufonamides	2 (1%)	0 (0%)	2 (1%)	0 (0%)	
Glycopeptides (vancomycin)	59 (23%)	19 (30%)	29 (21%)	1 (10%)	
Others	39 (18%)	6 (10%)	27 (19%)	6 (60%)	

The median time from the index surgery to I&D was 21 days (Table 5). There were 123 patients (92%) who required I&D within 90 days of index surgery (early presentation) and 12 patients who required I&D after 90 days (late presentation). Among the early-presenting subjects were 41 of 42 (99%) non-instrumented subjects, 81 of 87 (93%) instrumented-retained subjects, and one of six (17%) instrumented-removed subjects. Among the late-presenting subjects were one of 42 (1%) non-instrumented subjects, six of 87 (7%) instrumented-retained subjects, and five of six (83%) instrumented-removed subjects. Thus, the instrumented-removed subjects predominately presented late, but the other groups predominately presented early. (Interestingly, the instrumented-removed subjects also had a significantly higher incidence of prior MRSA infection (Table 2), but we were unable to study this further in our study.) There was no statistical difference between the non-instrumented, instrumented-removed, and instrumented-removed cohorts with respect to the persistence of infection (p = 0.80, chi-square analysis).

**Table 5:** Irrigation and debridement details I&D: irrigation and debridement, VAC: vacuum-assisted closure

Variable	All patients (n = 135)	Non-instrumented (n = 42)	Instrumented-retained (n = 87)	Instrumented-removed (n = 6)	p-value
Interval between the index surgery and I&D (days), median (range)	21 (5-2758)	17 (8-241)	22 (5-2758)	186 (40-340)	<0.01
Persistent infection	9 (7%)	3 (7%)	6 (7%)	0 (0%)	0.80
Number of I&D, n (%)					
One, 51 (38%)	51 (38%)	23 (55%)	27 (31%)	1 (17%)	0.03
Two, 58 (43%)	58 (43%)	13 (31%)	43 (49%)	2 (33%)	
Three or more, 26 (19%)	26 (19%)	6 (13%)	17 (20%)	3 (50%)	
Debridement technique, n (%)					
Primary wound closure	50 (37%)	23 (55%)	26 (30%)	1 (17%)	0.01
VAC therapy	85 (63%)	19 (45%)	61 (70%)	5 (83%)	

Fifty of the 135 patients (37%) received I&D and primary wound closure, and 85 of the 135 (63%) patients received I&D and VAC treatment prior to closure. Factors we considered included the amount of myonecrosis, the amount of dirty granulation tissue, the appearance of pus, the timing of patient presentation after index surgery, and the presence of signs of sepsis. Significantly more of the non-instrumented subjects received primary wound closure compared to the instrumented-retained/removed subjects, and significantly more of the instrumented-retained/removed patients received VAC therapy compared to the non-instrumented patients (p = 0.01). None of the patients who underwent primary wound closure subsequently required VAC placement. The average number of debridements was 2. When we stratified patients receiving VAC treatment into those who received one or two I&D and those who received three or more I&D, we observed no significant statistical difference (p = 0.52).

Retention of implants

At the first I&D, 42 patients had no implants and 93 had implants present. There was no difference in the subsequent rate of persistent infection whether implants were present or not at the first I&D (p = 0.80). Six of the 93 patients with implants present underwent implant removal at the first I&D. Therefore, following the first I&D, 48 (36%) patients had no implants present versus 87 patients (64%) retained implants. The persistent infection rate for the implant retention patients (instrumented-retained group) was 7% (6/87) and that of the no-implant patients (non-instrumented plus instrumented-removed groups) was 6% (3/48).

Persistent infection and pseudoarthrosis

Eighty-five (85) patients had attempted fusion as a part of their index surgery (Table 3): six had persistent infections and 79 patients had no persistent infection. Of the six with a persistent infection, one (17%) was identified with pseudoarthrosis. Of the 79 with no persistent infections, 13 (16%) were identified with pseudoarthrosis. Therefore, those with a persistent infection were no more likely to exhibit pseudoarthrosis than those with no persistent infection. Overall, pseudoarthrosis in infected patients with attempted fusion was 16% (14/85).

Patient-reported outcomes

**Figure 2:**
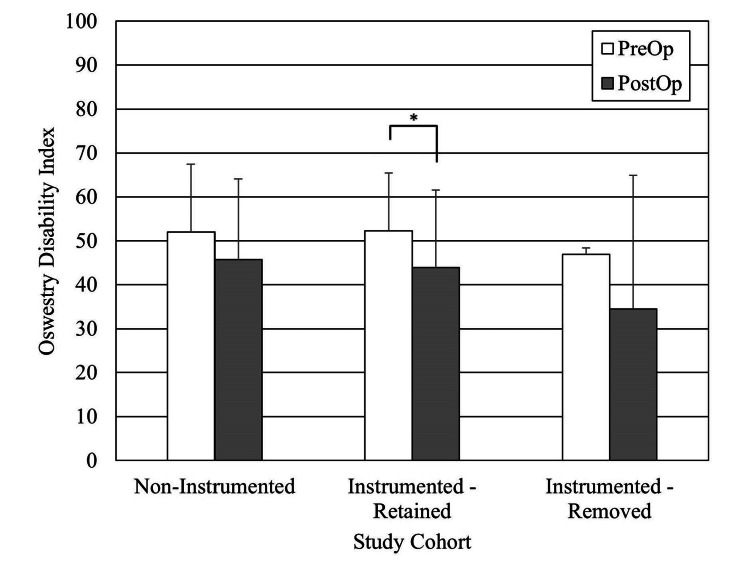
Pre- and postoperative Oswestry Disability Index (ODI) results for the no-persistent infection and persistent infection cohorts, minimum one-year follow-up. (*) Statistically significantly different

**Figure 3:**
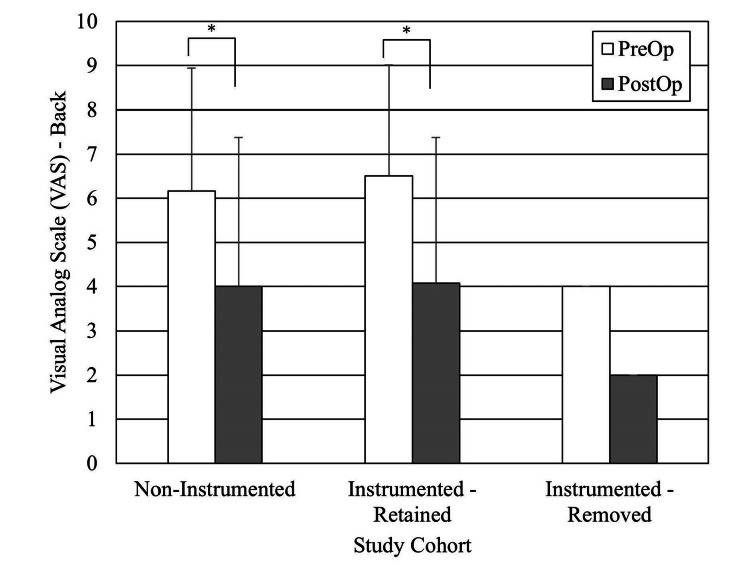
Pre- and postoperative Visual Analog Scale (VAS) Back results for the no-persistent infection and persistent infection cohorts, minimum one-year follow-up. (*) Statistically significantly different

**Figure 4:**
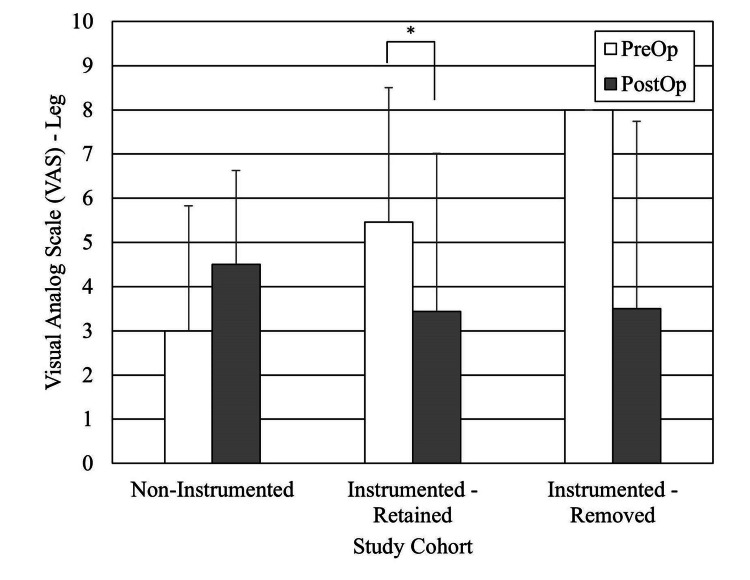
Pre- and postoperative Visual Analog Score (VAS) Leg results for the no-persistent infection and persistent infection cohorts, minimum one-year follow-up. (*) Statistically significantly different

For the outcomes analysis, we excluded the 17 patients who received cervical, cervicothoracic, and thoracic spine surgery patients as the subpopulation was too small to study. Of the remaining 118 subjects (thoracolumbar, lumbar, and sacral spine surgery patients), 14 were deceased at the time of study. We also excluded subjects who did not have preoperative scores or postoperative follow-up less than one year. There were 64 subjects with Oswestry Disability Index (ODI) scores, 55 with VAS back scores, and 53 with VAS leg scores (Figures 2, 3, 4). The average follow-up for these patient-reported outcomes was 2.7 years (range, 1.0-11.7 years). All improved significantly. The ODI MCID was achieved in 33% of the patients, VAS back in 42%, and VAS leg pain in 53% of patients. When stratified for implants present versus no implants present or persistent infection versus no persistent infection, there were no differences in the proportions of patients achieving MCID (Table 6). Achieving an MCID in ODI, VAS back, or VAS leg did not depend upon the implant status or persistent infection status.

**Table 6:** MCID achievement, minimum one-year follow-up Note: MCID values were 12.8 for ODI, 1.2 for VAS-Back, and 1.6 for VAS-Leg. MCID: minimum clinically important difference, ODI: Oswestry Disability Index, VAS: Visual Analog Scale

Patient-reported outcome	All patients	Non-instrumented	Instrumented-retained	Instrumented-removed	p-value
ODI (n = 64)	43/64 (67%)	11/17 (33%)	31/45 (69%)	1/2 (50%)	0.83
VAS-Back (n = 55)	31/55 (56%)	8/15 (53%)	22/39 (56%)	1/1 (100%)	0.66
VAS-Leg (n = 53)	25/53 (47%)	2 (100%)	22/49 (45%)	1/2 (50%)	0.31

## Discussion

Núñez-Pereira et al. evaluated the survival of spinal implants after postoperative DWI [10]. ROIs were required in 23% of the patients because of the lack of control of infection and the persistence of infection. Kowalski et al. performed a retrospective cohort study to evaluate risk factors for failure of treatment in patients with early and late onsets of infections in 81 patients [11]. They reported 35% failure of treatment with I&D and implant retention for early onset of DWI (less than 30 days) and 54% failure of treatment with I&D and implant retention for late onset of DWI (>30 days). By contrast, we demonstrated a 7% persistent infection rate with I&D and implant retention for the early onset of DWI (less than 90 days) and 0% for the late onset of DWI (>90 days). Our late-onset results are consistent with those of Khanna et al., who reported in a long-term follow-up study of 67 patients that none of their patients with retained implants had a recurrence of infection [6]. One possible explanation for the difference between our series and those of Núñez-Pereira et al. and Kowalski et al. is that the I&D treatment differed between the studies. Núñez-Pereira et al. and Kowalski et al. relied solely upon antibiotic treatment therapies, whereas we employed VAC treatment in 63% of cases (85/135).

Núñez-Pereira et al. found the number of levels fused, fusion to the pelvis, the number of debridements, and late presentation (>12 days) to be statistically significant risk factors for having implants removed [10]. We did not find any significant patient-specific or surgery-specific risk factors, perhaps because our persistent infection rate was much lower in patients with retained implants (7%). In our study, there was no statistical difference between early-onset and late-onset infections for persistent infection rate with implant retention (p = 1.0). All our patients with late onset of infection or DWI (>90 days) were able to keep their implants.

Núñez-Pereira et al. reported that 45% of patients who underwent ROI as a part of treatment for DWI require re-instrumentation for loss of correction or lack of fusion [10]. Kowalski et al. reported that 30% of patients required re-instrumentation due to various reasons [11]. Thus, the literature suggests that 30-45% of patients who undergo ROI for the management of DWI will require re-instrumentation.

This study showed that primary closure versus closure after VAC treatment had no impact on the successful treatment of DWI. However, we suspect selection bias as primary wound closure was used in “less serious” cases, and VAC treatment was used in “more serious” cases. We collected data about the types of pathogens, but there was no difference between the persistent infection and no persistent infection groups. Overall, we found a 9.4% persistent infection rate (eight of 85) with VAC treatment therapy. This agrees with Ploumis et al., who reported a 7.5% persistent infection rate with VAC treatment without implant removal in another study from our center [12]. However, when a patient underwent two I&Ds, the rate was 13.5%, and when a patient underwent three or more I&Ds, the rate was 0%. This suggests that premature closure of the wound after VAC treatment should be avoided. When a surgeon decided VAC treatment was necessary for DWI, more than two I&Ds resulted in a significantly better cure for the infection.

The literature is mixed for patient-reported outcomes of spinal surgery after postoperative DWI [13]. Petilon et al. performed a propensity score-matched case-control study to compare the two-year outcomes of 30 patients who received instrumented spinal fusion complicated by DWI with 30 patients who received instrumented spinal fusion without DWI [14]. ODI, SF-36 PCS, and VAS back and leg pain scores were significantly better in the follow-up in both groups. Moreover, patients with DWI have greater back pain and a decreased probability of achieving MCID for ODI than patients without. By contrast, Mok et al. published a retrospective case-control study comparing 16 patients with DWI after instrumented spinal fusion versus 16 patients without [15]. There were no significant differences in the physical component of the Medical Outcomes Study SF-36 general health survey at the two-year follow-up. Our study reports that all patients had statistically significant improvement in patient-reported outcomes, like ODI, VAS back pain, and VAS leg pain at minimum one-year follow-up.

Limitations

A potential bias of this study was whether the presence of instrumentation at the time of primary I&D was the deciding factor when proceeding with primary closure of wound or wound VAC. Of the 42 patients with no instrumentation prior to the first I&D, 23 were primary wound closure and 19 were wound VAC. Of the 93 patients with instrumentation prior to the first I&D, 27 were primary wound closure and 66 were wound VAC. Therefore, about half of the patients going into their first I&D with no instrumentation ended up getting primary wound closure. About one-third of patients going into their first I&D with instrumentation ended up getting primary wound closure. This difference is statistically significant (p < 0.01). Post primary I&D, 48 patients had no instrumentation (42 had no instrumentation and six had instrumentation removed), and 87 patients had instrumentation. Proportionally, more patients with instrumentation present also received wound VAC treatment compared to patients with no instrumentation (p < 0.02). Random assignment of patients into primary closure or wound VAC cohorts in a prospective clinical trial may avoid this source of bias. There were differences in surgical approach, objective, type of graft used, blood loss, and length of surgery, among others. These differences are potential confounders that might have created study bias. Another potential bias was that patient-reported outcomes were missing or incomplete for some subjects, and we had too few cervical, cervicothoracic, and thoracic subjects for the outcomes analysis. Lastly, because this is a single-center study at a specialized spine unit, the results may not be generalizable to other types of institutions.

## Conclusions

There was no difference in persistent infections between patients with implants present and those with no implants present. There was no identifiable risk factor for persistent infections in demographic data, health history data, and index surgical data. Comparison between primary closure of wound and closure after VAC treatment showed no impact on successful treatment of DWI. VAC treatment results are better with more than two I&Ds, suggesting that when wounds are “needing” debridement with the VAC therapy, avoiding premature closure of the wound can prevent persistent infections. Those with a persistent infection were no more likely to exhibit pseudoarthrosis than those with no persistent infection, but all groups had a higher pseudoarthrosis than non-infected cohorts in the literature. Despite DWIs, patients showed statistically significant improvement in ODI, VAS leg, and VAS back pain at a minimum one-year follow-up (average 2.7 years follow-up). In our study, we found that the removal of implants was not required to successfully treat DWIs after spine surgery.
